# Supplementary material for: Content-rich biological network constructed by mining PubMed abstracts
Source: BMC Bioinformatics. 2004 Oct 8;5:147. doi: 10.1186/1471-2105-5-147 (PMC528731; doi:10.1186/1471-2105-5-147)
Supplement: Additional File 2 — The original results of the above study (non-essential files are deleted to keep the file size under the limit set by BMC bioinformatics). [file 1471-2105-5-147-S2.bz2 › chilibotAdditionalFile2/dip40/302ID8647433E1293/html/U2AF1_U2AF2.html]

 


 **U2AF1** and **U2AF2** 
  
Found 123 abstracts in PubMed, retrieved 40.  
 

 What does Google say? 
 PDF only 
| .edu only 

---

**Interactive relationship** (e.g. stimulation, inhibition, etc)

**Neutral relationship**- The splicing factor U2AF  [ **U2AF2** ]   [ **U2AF1** ]  65, U2 small nuclear ribonucleoprotein particle snRNP auxillary factor of 65 kDa, binds to pyrimidine rich sequences at 3 splice sites to recruit U2 snRNP to pre mRNAs.  Ref: 12558503 Biochem J, 2003
- Yeast two hybrid analysis and immunoprecipitation indicated interaction of ZNF265 with the essential splicing factor proteins U1 70K and U2AF  [ **U2AF2** ]  35  [ **U2AF1** ] .  Ref: 11448987 J Cell Biol, 2001
- In this model, recruitment requires interactions between the SR proteins and the 35 kDa subunit of U2AF  [ **U2AF2** ]   [ **U2AF1** ]  U2AF35  [ **U2AF1** ] .  Ref: 11421359 RNA, 2001
- We propose that hnRNP H binds to the HIV 1 ESS2p element and competes with U2AF  [ **U2AF2** ]  35  [ **U2AF1** ]  for binding to the exon sequence flanking 3 splice site A3.  Ref: 11526107 J Biol Chem, 2001
- Complementary biochemical experiments demonstrate that the core U2AF  [ **U2AF2** ]   [ **U2AF1** ]  heterodimer binds RNA,.  Ref: 11551507 Cell, 2001
- SF2 ASF binds to a splicing enhancer in the third HIV 1 tat exon and stimulates U2AF  [ **U2AF2** ]   [ **U2AF1** ]  binding independently of the RS domain.  Ref: 11575921 J Mol Biol, 2001
- It has been proposed that the physical interaction between the arginine serine rich RS domain of U2AF  [ **U2AF2** ]  35  [ **U2AF1** ]  and SR proteins is important for this activity.  Ref: 11739736 Mol Cell Biol, 2002
- Copurification of the recombinant U2AF35  [ **U2AF1** ]  RNA recognition motif U2AF35  [ **U2AF1** ]  RRM and full length U2AF65  [ **U2AF2** ]  yields a soluble and functionally active minimal U2AF  [ **U2AF2** ]   [ **U2AF1** ]  heterodimer.  Ref: 12297299 FEBS Lett, 2002
- Here, we show that the pyrimidine tract of the last splice acceptor site of the human beta globin gene is necessary to stimulate mRNA 3 end formation in vivo and binds the U2AF  [ **U2AF2** ]   [ **U2AF1** ]  65 splicing factor.  Ref: 12189174 EMBO Rep, 2002
- Several lines of evidence indicate that the SL RNP proteins function by participating in a trans splicing specific network of protein protein interactions analogous to the U1 snRNP SF1 BBP U2AF  [ **U2AF2** ]   [ **U2AF1** ]  complex that comprises the cross intron bridge in cis splicing.  Ref: 12050671 Nature, 2002
- The first 60 nucleotides of the major element have 68% identity to the negative regulatory element of HPV 16, and these elements bind the same cellular proteins, CstF 64, U2AF  [ **U2AF2** ]   [ **U2AF1** ]  65, and HuR.  Ref: 12021332 J Virol, 2002
- Specifically, PTB binds polypyrimidine rich intronic elements upstream of alternatively spliced exons to antagonize the binding of the essential U2AF  [ **U2AF2** ]   [ **U2AF1** ]  splicing factor and repress the use of the regulated exons in specific tissues RNA, 1 1995 234.  Ref: 11804786 Mech Dev, 2002
- It has been shown that the interaction between U2AF  [ **U2AF2** ]  35  [ **U2AF1** ]  and the 3 splice site AG can stabilize U2AF  [ **U2AF2** ]   [ **U2AF1** ]  65 binding to weak Py tracts characteristic of so called AG dependent pre mRNAs.  Ref: 11604503 Mol Cell Biol, 2001
- We identified four RNA splicing factors hnRNP C, U2AF  [ **U2AF2** ]   [ **U2AF1** ]  U2 auxiliary factor, PTB polypyrimidine tract binding protein, and PSF PTB associated splicing factor that bind to two short regions 3 of the CUG n, and found a novel 3 DMPK exon resulting in an mRNA lacking the repeats.  Ref: 10911990 Mol Cell, 2000
- The U2 small nuclear ribonucleoprotein auxiliary factor  [ **U2AF2** ]   [ **U2AF1** ]  U2AF  [ **U2AF2** ]   [ **U2AF1** ]  is a heterodimeric splicing factor composed of 65 kDa U2AF  [ **U2AF2** ]   [ **U2AF1** ]  65 and 35 kDa U2AF  [ **U2AF2** ]  35  [ **U2AF1** ]  subunits.  Ref: 11118443 J Biol Chem, 2001
- The essential splicing factor U2AF  [ **U2AF2** ]   [ **U2AF1** ]  U2 auxiliary factor is a heterodimer composed of 65 kDa U2AF  [ **U2AF2** ]   [ **U2AF1** ]  65 and 35 kDa U2AF  [ **U2AF2** ]  35  [ **U2AF1** ]  subunits.  Ref: 11739736 Mol Cell Biol, 2002
- We found that the recombinant U2AF35  [ **U2AF1** ]  RRM is unstructured in solution but its tertiary structure is induced upon binding to U2AF65  [ **U2AF2** ] .  Ref: 12297299 FEBS Lett, 2002
- Collectively, the results argue that interaction of U2AF  [ **U2AF2** ]  35  [ **U2AF1** ]  with a consensus 3 splice site triggers events in spliceosome assembly in addition to stabilizing U2AF  [ **U2AF2** ]   [ **U2AF1** ]  65 binding, thus revealing a dual function for U2AF  [ **U2AF2** ]  35  [ **U2AF1** ]  in pre mRNA splicing.  Ref: 11604503 Mol Cell Biol, 2001
- In contrast, RS domain mediated interactions with SR proteins bound to the ESE appear to be dispensable, because the purine rich ESE present in exon M2 is not essential for U2AF  [ **U2AF2** ]  35  [ **U2AF1** ]  activity and because a truncation mutant of U2AF  [ **U2AF2** ]  35  [ **U2AF1** ]  consisting only of the pseudo RNA recognition motif domain and lacking the RS domain is active in our complementation assays.  Ref: 11604503 Mol Cell Biol, 2001
- We further found in human cells that the exogenously expressed large U2AF  [ **U2AF2** ]   [ **U2AF1** ]  subunit, U2AF65  [ **U2AF2** ] , accumulates in spliced mRNP, leading to the recruitment of U2AF35  [ **U2AF1** ]  and TAP.  Ref: 11724776 J Biol Chem, 2002
- Sex lethal splicing autoregulation in vivo interactions between SEX LETHAL, the U1 snRNP and U2AF  [ **U2AF2** ]   [ **U2AF1** ]  underlie male exon skipping.  Ref: 12490553 Development, 2003
- Complementation of the splicing activity of nuclear extracts depleted of U2AF  [ **U2AF2** ]   [ **U2AF1** ]  by chromatography in oligo dT cellulose requires, for some pre mRNAs, only the presence of U2AF  [ **U2AF2** ]   [ **U2AF1** ]  65.  Ref: 11604503 Mol Cell Biol, 2001
- Although U2AF  [ **U2AF2** ]   [ **U2AF1** ]  65 binding was reduced in the depleted extracts, the defects caused by SF1 mBBP depletion could not be fully restored by an increase in occupancy of the Py tract by exogenously added U2AF  [ **U2AF2** ]   [ **U2AF1** ]  65, arguing for a role of SF1 mBBP in U2 snRNP recruitment distinct from promoting U2AF  [ **U2AF2** ]   [ **U2AF1** ]  65 binding.  Ref: 10954700 J Biol Chem, 2000
- The 65 kDa subunit of U2AF  [ **U2AF2** ]   [ **U2AF1** ]  U2AF  [ **U2AF2** ]   [ **U2AF1** ]  65 binds to the polypyrimidine Py tract preceding the 3 splice site, while the 35 kDa subunit U2AF  [ **U2AF2** ]  35  [ **U2AF1** ]  contacts the conserved AG dinucleotide at the 3 end of the intron.  Ref: 11604503 Mol Cell Biol, 2001
- Finally, we show that U2AF  [ **U2AF2** ]   [ **U2AF1** ]  26 functions by enhancing the binding of U2AF  [ **U2AF2** ]   [ **U2AF1** ]  65 to weak 3 splice sites.  Ref: 11739736 Mol Cell Biol, 2002
- Second, U2AF  [ **U2AF2** ]  35  [ **U2AF1** ]  is thought to play a role in the recruitment of U2AF  [ **U2AF2** ]   [ **U2AF1** ]  65 by serine arginine rich SR proteins in enhancer dependent splicing.  Ref: 11739736 Mol Cell Biol, 2002

**Non-interactive relationship** (e.g. studied together, co-existance, homology, etc.)

- Some experiments support a model in which serine arginine SR rich proteins function as splicing activators by binding to enhancers and recruiting the splicing factor U2AF  [ **U2AF2** ]   [ **U2AF1** ]  to an adjacent weak 3 splice site.  Ref: 11421359 RNA, 2001
- , Cell 100 323 33 000, we propose a plausible arrangement for SF1 mBBP U2AF  [ **U2AF2** ]   [ **U2AF1** ]  complexes assembled at 3 splice sites.  Ref: 11438677 Mol Cell Biol, 2001
- The X ray structure of the human core U2AF  [ **U2AF2** ]   [ **U2AF1** ]  heterodimer, consisting of the U2AF35  [ **U2AF1** ]  central domain and a proline rich region of U2AF65  [ **U2AF2** ] , has been determined at 2.2 A resolution.  Ref: 11551507 Cell, 2001
- The human 56 kD U2AF  [ **U2AF2** ]   [ **U2AF1** ]  65 associated protein hUAP56, a member of the DExD H box protein family of RNA dependent ATPases, is required for the stable binding of U2 snRNP to the pre mRNA branchpoint.  Ref: 11156602 Genes Dev, 2001
- A novel function for the U2AF  [ **U2AF2** ]   [ **U2AF1** ]  65 splicing factor in promoting pre mRNA 3 end processing.  Ref: 12189174 EMBO Rep, 2002
- Several lines of evidence strongly suggest that the increased binding of U2 snRNP is mediated by U2AF  [ **U2AF2** ]   [ **U2AF1** ] .  Ref: 11421357 RNA, 2001
- Here, we report the identification of a mammalian gene that encodes a 26 kDa protein bearing strong sequence similarity to U2AF  [ **U2AF2** ]  35  [ **U2AF1** ] , designated U2AF  [ **U2AF2** ]   [ **U2AF1** ]  26.  Ref: 11739736 Mol Cell Biol, 2002
- The S.pombe SF1 BBP, U2AF  [ **U2AF2** ]   [ **U2AF1** ]  59 and U2AF  [ **U2AF2** ]   [ **U2AF1** ]  23 are tightly associated in a novel complex that is required for complex A formation.  Ref: 12374752 EMBO J, 2002
- The role of U2AF35  [ **U2AF1** ]  and U2AF65  [ **U2AF2** ]  in enhancer dependent splicing.  Ref: 11421359 RNA, 2001
- In the present study we determined the role of RS domains in the subcellular localization of U2AF  [ **U2AF2** ]   [ **U2AF1** ] .  Ref: 11118443 J Biol Chem, 2001
- Surprisingly, depletion of hnRNP A1 from the nuclear extract NE enables splicing to proceed in NE that contains 100 fold reduced concentrations of U2AF  [ **U2AF2** ]   [ **U2AF1** ]  and normal levels of SR proteins, conditions that do not support processing of other efficiently spliced pre mRNAs.  Ref: 11598017 EMBO J, 2001
- The results indicate that i the IgM substrate is an AG dependent pre mRNA, ii U2AF  [ **U2AF2** ]  35  [ **U2AF1** ]  dependence correlates with AG dependence, and iii the identity of the first nucleotide of exon 2 is important for U2AF  [ **U2AF2** ]  35  [ **U2AF1** ]  function.  Ref: 11604503 Mol Cell Biol, 2001
- These studies identify U2AF  [ **U2AF2** ]   [ **U2AF1** ]  26 as a mammalian splicing factor and demonstrate that distinct U2AF  [ **U2AF2** ]   [ **U2AF1** ]  complexes can participate in pre mRNA splicing.  Ref: 11739736 Mol Cell Biol, 2002
- Mutations in the large subunit of U2AF  [ **U2AF2** ]   [ **U2AF1** ]  disrupt pre mRNA splicing, cell cycle progression and nuclear structure.  Ref: 10923022 Yeast, 2000
- The RS domain on either U2AF  [ **U2AF2** ]   [ **U2AF1** ]  65 or U2AF  [ **U2AF2** ]  35  [ **U2AF1** ]  acts as a nuclear localization signal and is sufficient to target a heterologous protein to the nuclear speckles.  Ref: 11118443 J Biol Chem, 2001
- Therefore, we propose that U2AF  [ **U2AF2** ]   [ **U2AF1** ]  65 promotes 3 end processing, which contributes to 3 terminal exon definition.  Ref: 12189174 EMBO Rep, 2002
- Moreover, because U2AF  [ **U2AF2** ]   [ **U2AF1** ]  and the U1 snRNP are only associated transiently with the pre mRNA during the course of spliceosome assembly, our studies are difficult to reconcile with the current model that proposes that the SEX LETHAL blocks splicing at the second catalytic step, and instead argue that the SEX LETHAL protein acts after splice site recognition, but before catalysis begins.  Ref: 12490553 Development, 2003
- Like several other proteins involved in constitutive and regulated splicing, both U2AF  [ **U2AF2** ]   [ **U2AF1** ]  65 and U2AF  [ **U2AF2** ]  35  [ **U2AF1** ]  contain an arginine serine rich RS domain.  Ref: 11118443 J Biol Chem, 2001
- Using yeast two hybrid screens and in vitro binding studies, we found that this region coincides with a direct binding site for U2AF35  [ **U2AF1** ] , the small subunit of the splicing factor U2AF  [ **U2AF2** ]   [ **U2AF1** ] .  Ref: 11724776 J Biol Chem, 2002
- Using antibodies to the splicing factors PSF, U2AF  [ **U2AF2** ]   [ **U2AF1** ]  65 and snRNPs we find that these proteins localize in foci throughout the nuclei of immature bone marrow cells.  Ref: 11255221 J Cell Biochem, 2001
- The present work shows that, in addition to the previously identified participants, the ratio of exon 6 isoforms is affected by 1 suppression of the cryptic sites, 2 deletions of the upstream intron, and 3 the splicing regulators PTB and U2AF  [ **U2AF2** ]   [ **U2AF1** ] , both of which act on the branch point polypyrimidine tract region.  Ref: 11038253 Brain Res Mol Brain Res, 2000
- The fission yeast ortholog of the coregulator SKIP interacts with the small subunit of U2AF  [ **U2AF2** ]   [ **U2AF1** ] .  Ref: 11414703 Biochem Biophys Res Commun, 2001
- U2AF  [ **U2AF2** ]  35  [ **U2AF1** ]  has also been implicated in arginine serine RS domain mediated bridging interactions with splicing factors of the SR protein family bound to exonic splicing enhancers ESE, and these interactions can also stabilize U2AF  [ **U2AF2** ]   [ **U2AF1** ]  65 binding.  Ref: 11604503 Mol Cell Biol, 2001
- Pop3 Wat1 has also been isolated from a two hybrid screen as a binding partner to Prp2, the large subunit of the essential splicing factor U2AF  [ **U2AF2** ]   [ **U2AF1** ] .  Ref: 11686295 J Cell Sci, 2001
- The arginine and serine rich domain of U2AF  [ **U2AF2** ]   [ **U2AF1** ]  65 is critical for U1 recruitment, and we discuss the role of its RNA RNA annealing activity in this novel function of U2AF  [ **U2AF2** ]   [ **U2AF1** ]  65.  Ref: 12558503 Biochem J, 2003
- Structural Basis for the Molecular Recognition between Human Splicing Factors U2AF  [ **U2AF2** ]   [ **U2AF1** ]  65 and SF1 mBBP.  Ref: 12718882 Mol Cell, 2003
- The structure of the C terminal RRM RRM3 of human U2AF  [ **U2AF2** ]   [ **U2AF1** ]  65 complexed to an N terminal peptide of SF1 reveals an extended negatively charged helix A and an additional helix C.  Ref: 12718882 Mol Cell, 2003
- Recombinant U2AF35  [ **U2AF1** ]  RRM protein free and in complex with three different regions of U2AF65  [ **U2AF2** ]  was characterized by nuclear magnetic resonance spectroscopy.  Ref: 12297299 FEBS Lett, 2002
- Shuttling of U2AF  [ **U2AF2** ]   [ **U2AF1** ]  between nucleus and cytoplasm possibly represents a means to control the availability of this factor to initiate spliceosome assembly and therefore contribute to regulate splicing.  Ref: 11118443 J Biol Chem, 2001
- The structure reveals a novel protein protein recognition strategy, in which an atypical RNA recognition motif RRM of U2AF35  [ **U2AF1** ]  and the U2AF65  [ **U2AF2** ]  polyproline segment interact via reciprocal tongue in groove tryptophan residues.  Ref: 11551507 Cell, 2001
- We have previously shown that SXL blocks the binding of U2 auxiliary factor U2AF  [ **U2AF2** ]   [ **U2AF1** ]  to the polypyrimidine Py tract associated with the 3 splice site of the regulated intron.  Ref: 11565743 RNA, 2001
- Most importantly, we find that splicing activators promote the binding of both U2AF65  [ **U2AF2** ]  and U2AF35  [ **U2AF1** ]  to weak 3 splice sites under splicing conditions.  Ref: 11421359 RNA, 2001
- First, U2AF  [ **U2AF2** ]  35  [ **U2AF1** ]  has been shown to function by directly interacting with the AG at the 3 splice site.  Ref: 11739736 Mol Cell Biol, 2002
- The structural basis for recognition between human splicing factors U2AF  [ **U2AF2** ]   [ **U2AF1** ]  65 and SF1 mBBP has recently been elucidated, setting the scene for understanding a key step in pre mRNA splicing and revealing a new conserved function for RRM proteins.  Ref: 12737811 Structure Camb , 2003
- Pre spliceosome formation in S.pombe requires a stable complex of SF1 U2AF  [ **U2AF2** ]   [ **U2AF1** ]  59 U2AF  [ **U2AF2** ]   [ **U2AF1** ]  23.  Ref: 12374752 EMBO J, 2002
- U2 auxiliary factor U2AF  [ **U2AF2** ]   [ **U2AF1** ]  is an essential splicing factor that recognizes the 3 splice site and recruits the U2 snRNP to the branch point.  Ref: 11551507 Cell, 2001
- The RRM3 of U2AF  [ **U2AF2** ]   [ **U2AF1** ]  65 shows no cross linking to the Py tract.  Ref: 12554879 RNA, 2003
- The splicing factors and the transcription machinery co purify through a gel filtration column and co immunoprecipitate in experiments using an anti U2AF  [ **U2AF2** ]   [ **U2AF1** ]  65 antibody, indicating that they are part of a unique complex.  Ref: 11773074 J Biol Chem, 2002
- Naturally occurring beta thalassaemia causing mutations within the pyrimidine tract reduces both U2AF  [ **U2AF2** ]   [ **U2AF1** ]  65 binding and 3 end cleavage efficiency.  Ref: 12189174 EMBO Rep, 2002
- Dual function for U2AF  [ **U2AF2** ]  35  [ **U2AF1** ]  in AG dependent pre mRNA splicing.  Ref: 11604503 Mol Cell Biol, 2001
- However, the C terminal domain of U2AF  [ **U2AF2** ]   [ **U2AF1** ]  26 lacks many characteristics of the U2AF  [ **U2AF2** ]  35  [ **U2AF1** ]  RS domain and, therefore, might be incapable of interacting with SR proteins.  Ref: 11739736 Mol Cell Biol, 2002
- Similar genetic and biochemical approaches also link SEX LETHAL with the heterodimeric splicing factor, U2AF  [ **U2AF2** ]   [ **U2AF1** ] .  Ref: 12490553 Development, 2003
- Kinetic role for mammalian SF1 BBP in spliceosome assembly and function after polypyrimidine tract recognition by U2AF  [ **U2AF2** ]   [ **U2AF1** ] .  Ref: 10954700 J Biol Chem, 2000
- We report that U2AF  [ **U2AF2** ]   [ **U2AF1** ]  65 can also promote the recruitment of U1 snRNP to weak 5 splice sites that are followed by uridine rich sequences.  Ref: 12558503 Biochem J, 2003
- Characterization of U2AF  [ **U2AF2** ]   [ **U2AF1** ]  6, a splicing factor related to U2AF  [ **U2AF2** ]  35  [ **U2AF1** ] .  Ref: 11739736 Mol Cell Biol, 2002
- We have used affinity chromatography with immobilized transcription elongation factor SII to purify a protein complex that contains core RNA polymerase II RNA Pol II, the general transcription initiation factors, and several splicing factors, including the U1, U2, and U4 small nuclear RNPs, the U2AF  [ **U2AF2** ]   [ **U2AF1** ]  65, and serine arginine rich proteins.  Ref: 11773074 J Biol Chem, 2002
- These results support the hypothesis that the IDDE facilitates the recognition of the 3 splice site preceding N30 by a network of protein protein interactions implicated in the recruitment of U2AF  [ **U2AF2** ]   [ **U2AF1** ]  to a suboptimal Py tract.  Ref: 10931847 J Biol Chem, 2000
- Furthermore, the results suggest that the presence of an RS domain in either U2AF  [ **U2AF2** ]   [ **U2AF1** ]  subunit is sufficient to trigger the nucleocytoplasmic import of the heterodimeric complex.  Ref: 11118443 J Biol Chem, 2001
- It inserts a conserved tryptophan into a hydrophobic pocket between helices A and B in a way that strikingly resembles part of the molecular interface in the U2AF  [ **U2AF2** ]   [ **U2AF1** ]  heterodimer.  Ref: 12718882 Mol Cell, 2003
- We show that U2AF  [ **U2AF2** ]   [ **U2AF1** ]  26 can associate with U2AF  [ **U2AF2** ]   [ **U2AF1** ]  65 and can functionally substitute for U2AF  [ **U2AF2** ]  35  [ **U2AF1** ]  in both constitutive and enhancer dependent splicing, demonstrating that the RS domain of the small U2AF  [ **U2AF2** ]   [ **U2AF1** ]  subunit is not required for splicing enhancer function.  Ref: 11739736 Mol Cell Biol, 2002
- We first established that SF1 mBBP and U2AF  [ **U2AF2** ]   [ **U2AF1** ]  cooperatively assemble in our reporter system at RNA sites composed of the BPS, PPT, and AG dinucleotide found at 3 splice sites, with endogenous proteins assembled along with the Tat fusions.  Ref: 11438677 Mol Cell Biol, 2001
- It also appears that some RNA processing factors are required for viability e.g., U2AF  [ **U2AF2** ]   [ **U2AF1** ] , PUF60, SRp54, SAP49, PRP8, U1 70K.  Ref: 12810918 RNA< MedlineTA>RNA, 2001
- The N terminal 187 amino acids of U2AF  [ **U2AF2** ]  35  [ **U2AF1** ]  and U2AF  [ **U2AF2** ]   [ **U2AF1** ]  26 are nearly identical.  Ref: 11739736 Mol Cell Biol, 2002
- This pre formed SF1 U2AF  [ **U2AF2** ]   [ **U2AF1** ]  59 U2AF  [ **U2AF2** ]   [ **U2AF1** ]  23 complex may represent a streamlined mechanism for recognition of the branch site, pyrimidine tract and 3 splice site at the 3 end of introns.  Ref: 12374752 EMBO J, 2002
- When the 3 splice site is weak, both the SF2 ASF RS domain and U2AF  [ **U2AF2** ]  35  [ **U2AF1** ]  are required for splicing.  Ref: 11124808 Genes Dev, 2000
- The acceptor exon nucleotides are part of the consensus required for the U2AF  [ **U2AF2** ]  35  [ **U2AF1** ]  mediated recognition of AG in such introns.  Ref: 11410667 Nucleic Acids Res, 2001
- To investigate how the KH domain of SF1 mBBP recognizes the BPS in conjunction with U2AF  [ **U2AF2** ]   [ **U2AF1** ]  and possibly other proteins, we constructed a transcriptional reporter system utilizing human immunodeficiency virus type 1 Tat fusion proteins and examined the RNA binding specificity of the complex using KH domain and RNA binding site mutants.  Ref: 11438677 Mol Cell Biol, 2001
- The requirements for S.pombe complex A formation pre mRNA sequence elements, protein splicing factors, SF1 BBP and both subunits of U2AF  [ **U2AF2** ]   [ **U2AF1** ]  are analogous to those of mammalian complex A.  Ref: 12374752 EMBO J, 2002
- Inspection of splice site consensus sequences shows that a polypyrimidine Py tract preceding N30 is suboptimal for U2AF  [ **U2AF2** ]   [ **U2AF1** ]  binding.  Ref: 10931847 J Biol Chem, 2000
- Both U2AF  [ **U2AF2** ]   [ **U2AF1** ]  65 and U2AF  [ **U2AF2** ]  35  [ **U2AF1** ]  are shown to shuttle continuously between the nucleus and the cytoplasm by a mechanism that involves carrier receptors and is independent from binding to mRNA.  Ref: 11118443 J Biol Chem, 2001
- The splicing factor U2AF  [ **U2AF2** ]   [ **U2AF1** ]  is required for the recruitment of U2 small nuclear RNP to pre mRNAs in higher eukaryotes.  Ref: 11604503 Mol Cell Biol, 2001
- First, we confirm that base substitutions that convert weak 3 splice sites to a consensus sequence, and therefore increase U2AF  [ **U2AF2** ]   [ **U2AF1** ]  binding, relieve the requirement for a splicing activator.  Ref: 11421359 RNA, 2001
- The human essential splicing factor U2AF  [ **U2AF2** ]   [ **U2AF1** ]  U2 auxiliary factor consists of 35 and 65 kDa subunits which form a highly stable heterodimer in solution.  Ref: 12297299 FEBS Lett, 2002
- Using the two hybrid screen, we examined the interaction partners of spSNW1, the S. pombe ortholog of the human coregulator SNW1 SKIP NCoA 62, and found it to interact with the small subunit of the splicing factor U2AF  [ **U2AF2** ]   [ **U2AF1** ]  spU2AF23.  Ref: 11414703 Biochem Biophys Res Commun, 2001
- We conclude that a critical function of splicing activators is to recruit U2AF  [ **U2AF2** ]   [ **U2AF1** ]  to the weak 3 splice sites of enhancer dependent introns,.  Ref: 11421359 RNA, 2001
- However, more recent experiments have not supported the U2AF  [ **U2AF2** ]   [ **U2AF1** ]  recruitment model.  Ref: 11421359 RNA, 2001
- Both RRM1 and RRM2 of U2AF  [ **U2AF2** ]   [ **U2AF1** ]  65 and SXL can be cross linked to certain residues, with RRM2 showing a surprisingly high number of residues cross linked.  Ref: 12554879 RNA, 2003
- Significantly, a fusion protein containing U2AF  [ **U2AF2** ]   [ **U2AF1** ]  65, when tethered upstream of a cleavage polyadenylation site, increases 3 end cleavage efficiency in vitro and in vivo.  Ref: 12189174 EMBO Rep, 2002
- The Drosophila splicing repressor Sex lethal SXL, which has two RRMs, can directly compete with the essential splicing factor U2AF  [ **U2AF2** ]   [ **U2AF1** ]  65, which has three RRMs, for binding to specific Py tracts.  Ref: 12554879 RNA, 2003
- U2AF  [ **U2AF2** ]  35  [ **U2AF1** ]  has multiple functions in pre mRNA splicing.  Ref: 11739736 Mol Cell Biol, 2002
- Reconstituting the extract with recombinant hnRNP A1 protein restores splicing inhibition at a step subsequent to U2AF  [ **U2AF2** ]   [ **U2AF1** ]  binding, mainly at the time of U2 snRNP association.  Ref: 11598017 EMBO J, 2001
- In contrast, splicing of a mouse immunoglobulin M IgM M1 M2 pre mRNA requires both U2AF  [ **U2AF2** ]   [ **U2AF1** ]  subunits.  Ref: 11604503 Mol Cell Biol, 2001
- U2AF  [ **U2AF2** ]   [ **U2AF1** ]  participates in the binding of TAP NXF1 to mRNA.  Ref: 11724776 J Biol Chem, 2002
- In contrast, proteins specific to earlier splicing complexes, such as U2AF  [ **U2AF2** ]   [ **U2AF1** ]  and U1 snRNP components, were not detected in C complex, but were present in similarly purified H complex.  Ref: 11991638 RNA, 2002
- Here we show that U2AF  [ **U2AF2** ]   [ **U2AF1** ]  interacts with the Py tract and downstream AG.  Ref: 12015979 Cell, 2002
- The large subunit of U2AF  [ **U2AF2** ]   [ **U2AF1** ]  recognizes the intronic polypyrimidine tract, a sequence located adjacent to the 3 splice site that serves as an important signal for both constitutive and regulated pre mRNA splicing.  Ref: 11118443 J Biol Chem, 2001
- In this report we have investigated the sequence elements e.g., Py tract strength, 3 splice site AG, ESE responsible for the U2AF  [ **U2AF2** ]  35  [ **U2AF1** ]  dependence of IgM.  Ref: 11604503 Mol Cell Biol, 2001
- The small subunit U2AF  [ **U2AF2** ]  35  [ **U2AF1** ]  interacts with the 3 splice site dinucleotide AG and is essential for regulated splicing.  Ref: 11118443 J Biol Chem, 2001
- Based on its sequence and functional similarity to U2AF  [ **U2AF2** ]  35  [ **U2AF1** ] , U2AF  [ **U2AF2** ]   [ **U2AF1** ]  26 may play a role in regulating alternative splicing.  Ref: 11739736 Mol Cell Biol, 2002
- Together with our finding that U2AF  [ **U2AF2** ]   [ **U2AF1** ]  is continuously exported from the nucleus, these data suggest that U2AF  [ **U2AF2** ]   [ **U2AF1** ]  participates in nuclear export, by facilitating TAP s addition to its mRNA substrates.  Ref: 11724776 J Biol Chem, 2002
- The essential splicing factors SF1 and U2AF  [ **U2AF2** ]   [ **U2AF1** ]  play an important role in the recognition of the pre mRNA 3 splice site during early spliceosome assembly.  Ref: 12718882 Mol Cell, 2003
- Nucleocytoplasmic shuttling of heterodimeric splicing factor U2AF  [ **U2AF2** ]   [ **U2AF1** ] .  Ref: 11118443 J Biol Chem, 2001
- While some of the effects of U2AF  [ **U2AF2** ]  35  [ **U2AF1** ]  can be explained in terms of enhanced U2AF  [ **U2AF2** ]   [ **U2AF1** ]  65 binding, other activities of U2AF  [ **U2AF2** ]  35  [ **U2AF1** ]  do not correlate with increased cross linking of U2AF  [ **U2AF2** ]   [ **U2AF1** ]  65 to the Py tract.  Ref: 11604503 Mol Cell Biol, 2001
- Induced folding of the U2AF35  [ **U2AF1** ]  RRM upon binding to U2AF65  [ **U2AF2** ] .  Ref: 12297299 FEBS Lett, 2002
- the interacting tryptophan side chains are essential for U2AF  [ **U2AF2** ]   [ **U2AF1** ]  dimerization.  Ref: 11551507 Cell, 2001
- Over expression of either TcSR or the human SR protein associated splicing factor splicing factor 2 ASF SF2 in wild type Schizosaccharomyces pombe, provoked an elongated phenotype similar to that of fission yeast over expressing the SR containing splicing factor Prp2, a U2AF  [ **U2AF2** ]   [ **U2AF1** ]  65 orthologue.  Ref: 12615334 Mol Biochem Parasitol, 2003
- A novel peptide recognition mode revealed by the X ray structure of a core U2AF35  [ **U2AF1** ]  U2AF65  [ **U2AF2** ]  heterodimer.  Ref: 11551507 Cell, 2001
- Sex lethal and U2 small nuclear ribonucleoprotein auxiliary factor  [ **U2AF2** ]   [ **U2AF1** ]  U2AF65  [ **U2AF2** ]  recognize polypyrimidine tracts using multiple modes of binding.  Ref: 12554879 RNA, 2003
- These results show the existence of an RS domain independent function of SR proteins in constitutive and enhancer dependent splicing, and suggest mechanisms for their role in enhancer function besides U2AF  [ **U2AF2** ]   [ **U2AF1** ]  recruitment.  Ref: 11124808 Genes Dev, 2000
- Initial recognition of these signals involves cooperative binding of the splicing factor SF1 mammalian branch point binding protein mBBP to the BP and of U2AF  [ **U2AF2** ]   [ **U2AF1** ]  65 to the Py tract.  Ref: 10954700 J Biol Chem, 2000
